# The neuropsychology of delirium: advancing the science of delirium assessment

**DOI:** 10.1002/gps.4711

**Published:** 2017-04-09

**Authors:** Zoë Tieges, Jonathan J. Evans, Karin J. Neufeld, Alasdair M.J. MacLullich

**Affiliations:** ^1^ Edinburgh Delirium Research Group University of Edinburgh Edinburgh UK; ^2^ Centre for Cognitive Ageing and Cognitive Epidemiology University of Edinburgh Edinburgh UK; ^3^ Institute of Health and Well Being University of Glasgow Glasgow UK; ^4^ Department of Psychiatry and Behavioral Sciences Johns Hopkins University School of Medicine Baltimore MD USA

**Keywords:** delirium, neuropsychological assessment, objective measurement, cognitive tests, attention, arousal

## Abstract

**Objective:**

The diagnosis of delirium depends on eliciting its features through mental status examination and informant history. However, there is marked heterogeneity in how these features are assessed, from binary subjective clinical judgement to more comprehensive methods supported by cognitive testing. The aim of this article is to review the neuropsychological research in delirium and suggest future directions in research and clinical practice.

**Methods:**

We reviewed the neuropsychological literature on formal assessment and quantification of the different domains in delirium, focusing on the core feature of inattention.

**Results:**

Few studies have characterised and quantified the features of delirium using objective methods commonly employed in neuropsychological research. The existing evidence confirms that patients with delirium usually show impairments on objective tests of attention compared with cognitively intact controls and, in most cases, compared with patients with dementia. Further, abnormal level of arousal appears to be a specific indicator of delirium. The neuropsychological evidence base for impairments in other cognitive domains in delirium, including visual perception, language and thought processes, is small.

**Conclusions:**

Delirium diagnosis requires accurate testing for its features, but there is little neuropsychological research examining the nature of these features, or evaluating the reliability, validity and discriminatory power of existing assessment processes. More research using the neuropsychological approach has enormous potential to improve and standardise delirium assessment methods of the individual features of delirium, such as inattention, and in developing more robust reference standards to enable greater comparability between studies.

## Introduction

The term delirium refers to a syndrome of cognitive, psychiatric and motor abnormalities that are commonly observed in acutely medically unwell patients, following surgery or trauma, or in the context of drug intoxication or withdrawal. The mental status changes arise rapidly, over hours to days, and often fluctuate. Most cases of delirium resolve within days, although around 20% persist for weeks or months (Cole, 2010). Several mental status abnormalities are typically considered to be part of the delirium syndrome. These include inattention, altered level of consciousness and cognitive deficits including memory, perception and language impairments. Current diagnostic criteria focus on inattention as the central feature of delirium (European Delirium Association and American Delirium Society, 2014); at least one other cognitive deficit and acute onset are also required to make the diagnosis (Table 1). Delirium characterisation in the major classification systems has shown some change over time (i.e. the Diagnostic Statistical Manual (DSM) (American Psychiatric Association (APA), 1980, 1987, 1994, 2013) and the International Classification of Diseases (ICD) (World Health Organization, 1992)).

**Table 1:** Diagnostic criteria for delirium listed in different classification systems: Diagnostic and Statistical Manual 3rd edition (DSM‐III; American Psychiatric Association, 1980), DSM‐III‐revised (American Psychiatric Association, 1987), DSM‐IV (American Psychiatric Association, 1994), DSM‐5 (American Psychiatric Association, 2013) and International Classification of Diseases 10th edition (World Health Organization, 1992)

Classification system	Diagnostic criteria for delirium
DSM‐III	A. Clouding of consciousness (reduced clarity of awareness of the environment), with reduced capacity to shift, focus and sustain attention to environmental stimuli
	B. At least two of the following: (1) perceptual disturbance; misinterpretations, illusions or hallucinations (2) speech that is at times incoherent (3) disturbance of sleep–wakefulness cycle, with insomnia or daytime drowsiness (4) increased or decreased psychomotor activity
	C. Disorientation and memory impairment (if testable)
	D. Clinical features that develop over a short period of time (usually hours to days) and tend to fluctuate over the course of a day
	E. Evidence, from the history, physical examination or laboratory tests, of a specific organic factor judged to be etiologically related to the disturbance
DSM‐III‐R	A. Reduced ability to maintain attention to external stimuli (e.g. questions must be repeated because attention wanders) and to appropriately shift attention to new external stimuli (e.g. perseverates answer to a previous question)
	B. Disorganised thinking, as indicated by rambling, irrelevant or incoherent speech
	C. At least two of the following: (1) reduced level of consciousness, e.g. difficulty keeping awake during examination (2) perceptual disturbances: misinterpretations, illusions or hallucinations (3) disturbance of sleep–wake cycle with insomnia or daytime sleepiness (4) increased or decreased psychomotor activity (5) disorientation to time, place or person (6) memory impairment, e.g. inability to learn new material, such as the names of several unrelated objects after 5 min or to remember past events, such as history of current episode of illness
	D. Clinical features develop over a short period of time (usually hours to days) and tend to fluctuate over the course of a day
	E. Either (1) or (2): (1) evidence from the history, physical examination or laboratory tests of a specific organic factor (or factors) judged to be etiologically related to the disturbance (2) in the absence of such evidence, an etiologic organic factor can be presumed if the disturbance cannot be accounted for by any nonorganic mental disorder, e.g. manic episode accounting for agitation and sleep disturbance
DSM‐IV	A. Disturbance of consciousness (i.e. reduced clarity of awareness of the environment) with reduced ability to focus, sustain or shift attention
	B. A change in cognition or the development of a perceptual disturbance that is not better accounted for by a pre‐existing, established or evolving dementia
	C. The disturbance develops over a short period of time (usually hours to days) and tends to fluctuate during the course of the day
	D. There is evidence from the history, physical examination or laboratory findings that the disturbance is caused by the direct physiological consequences of a general medical condition
DSM‐V	A. A disturbance in attention (i.e. reduced ability to direct, focus, sustain and shift attention) and awareness (reduced orientation to the environment)
	B. The disturbance develops over a short period of time (usually hours to a few days), represents a change from baseline attention and awareness and tends to fluctuate in severity during the course of a day
	C. An additional disturbance in cognition (e.g. memory deficit, disorientation, language, visuospatial ability or perception)
	D. The disturbances in Criteria A and C are not better explained by a pre‐existing, established or evolving neurocognitive disorder and do not occur in the context of a severely reduced level of arousal, such as coma
	E. There is evidence from the history, physical examination or laboratory findings that the disturbance is a direct physiological consequence of another medical condition, substance intoxication or withdrawal, or exposure to a toxin, or is due to multiple etiologies
ICD‐10	A. Clouding of consciousness: reduced clarity of awareness of the environment, with reduced ability to focus, sustain and shift attention
	B. Disturbance of cognition: both impairment of immediate recall and recent memory, with relatively intact remote memory, and disorientation in time, place or person
	C. Psychomotor disturbances: at least one of ‐Rapid, unpredictable shifts from hypo‐activity to hyper‐activity ‐Increased reaction time ‐Increased or decreased flow of speech ‐Enhanced startle reaction
	D. Disturbance of sleep–wake cycle: Manifest as ‐Insomnia, which in severe cases may involve total sleep loss, with or without daytime drowsiness, or reversal of sleep–wake cycle ‐Nocturnal worsening of symptoms ‐Disturbing dreams or nightmares, which may continue as hallucinations or illusions after waking
	E. Rapid onset and fluctuations of the symptoms over the course of the day
	F. Objective evidence from history, physical or neurological examination or laboratory tests of an underlying cerebral or systemic disease (other than psychoactive‐substance related) that can be presumed to be responsible for the clinical manifestations in A–D

Please note that these International Classification of Diseases (ICD)‐10 guidelines are diagnostic guidelines for the purpose of research, and other criteria are offered for clinical use.

Delirium is the product of many potential underlying aetiologies, and no biomarkers are currently used to assist diagnosis. Therefore, detection relies entirely on eliciting the key features, using a mixture of interview, cognitive testing, observation and informant history. However, there is little consensus on how these features are assessed, and a large number of methods is in use in clinical practice and research. These range from unstructured interviews followed by global clinical impression to more complex assessments using structured interviews and objective cognitive testing (Neufeld *et al.*, 2014). Notably, very few studies have attempted to compare methods of assessing the individual features of delirium. This means that, for example, we do not know if ‘inattention’ means the same in studies using different methods.

Good psychometric tests should show high reliability, high validity, good discriminatory power and extensive norms (Kline, 2000). There are several inter‐rater reliability studies of overall delirium diagnosis using various instruments and criteria and some studies examining validity and discriminatory power. Yet there are very few studies scrutinising assessments of the individual features of delirium with regard to the requirements of good tests. What evidence exists shows that inter‐rater reliability is often inadequate. For example, one study demonstrated marked variation in inter‐rater reliabilities for subjective ratings of delirium features by experienced clinicians (Kappa range 0.42–0.73 [acceptable to good reliability]) with lower numbers in patients with dementia (Kappa as low as 0.29 [questionable reliability]) (Sepulveda *et al.*, 2016). Further, inter‐rater agreement between subjective and objective approaches to delirium assessment in the intensive care unit (ICU) was found to vary considerably (Kappa range 0.62–0.93) (Guenther *et al.*, 2012).

Therefore, the science of delirium assessment remains very much a developing field. There is marked heterogeneity in how both the overall diagnosis and the determination of the presence or absence of individual features are carried out. Clearly, higher quality testing procedures and more consistency in how delirium is assessed would benefit the field; increasing adoption of a more formal neuropsychological approach as introduced in the following section has much to offer in addressing this need.

Neuropsychology is concerned with the relationship between brain and behaviour and in particular the behavioural expression of brain dysfunction (Lezak *et al.*, 2012). In relation to delirium, neuropsychology has the main aims of defining and accurately measuring the various features of delirium. Neuropsychological theorising and research has defined several major domains of cognition, such as attention, perception, memory, language and executive functions. Within each domain, sub‐domains or specific processes have been identified. A key task for clinical neuropsychology has been to develop methods of measuring these cognitive processes with tools that are reliable and valid and to provide a means of determining whether there is evidence of dysfunction. For many cognitive processes, there is a wide range of performance in the healthy population, which may be affected by many factors (e.g. age, gender and education). Therefore, determining whether a cognitive test score is ‘abnormal’ requires comparing the score with that from an appropriate reference group. Another important task for neuropsychology is to contribute to differential diagnosis of pathologies with similar or overlapping symptoms. In relation to delirium, the key differential diagnosis is with dementia, although other neuropsychiatric disorders including mood and psychotic disorders also share overlapping features. While one of the most important features differentiating these conditions is the nature of the onset (i.e. insidious or acute), the question arises as to whether the specific profile of cognitive deficits can also distinguish these conditions.

This article aims to provide a general introduction to neuropsychological research on delirium. We first review the neuropsychological evidence on formal assessment and quantification of the different symptom domains in delirium, then discuss implications and potential future directions of research.

We identified studies up to October 2016 published in English, which objectively examined a number of pre‐specified neuropsychological domains in delirium. We conducted electronic searches of MEDLINE and ISI Web of Knowledge databases; bibliographies of relevant articles and books were hand searched.

We included studies reporting on testing in patients with current delirium, excluding studies on delirium tremens and delirium in children. We only included studies that used DSM or ICD criteria for delirium or validated diagnostic methods on the basis of these criteria. This review does not cover all studies; rather, exemplars of relevant types of studies were selected.

## Attention

### Attention in delirium

The DSM‐5 criteria for delirium require that inattention is present, specifically that there is a ‘reduced ability to direct, focus, sustain and shift attention’(American Psychiatric Association, 2013; European Delirium Association and American Delirium Society, 2014). DSM‐5 also includes the constructs of ‘awareness’ and ‘orientation to the environment’, which relate both to the contents of consciousness and the level of arousal (LoA) (Table 1). Crucially, the guidance notes state that reduced LoA above the level of coma indicates severe inattention. This has implications for delirium assessment, because many patients with delirium are too drowsy to engage with cognitive testing or even interview (European Delirium Association and American Delirium Society, 2014).

Inattention is central to delirium, but it has been the subject of few neuropsychological studies in the context of delirium (Tieges *et al.*, 2014). In delirium research, inattention is mainly reported as being simply present or absent. Widely differing assessment methods are employed including binary subjective assessments following interview and performance on cognitive tests, although in the latter, score thresholds are mostly absent. Few studies have examined the reliability and validity of assessments of attention in delirium.

### Attention in psychology research

The multi‐component construct of attention has been extensively studied in psychology. Attention is commonly described as a preparedness for, and selection of, certain environmental or mental stimuli (Raz and Buhle, 2006). There is no agreed model of attention in psychology, with multiple inter‐related constructs described by various authors including focused, divided and executive attention, and so on. Nonetheless, there is some consensus that three major systems are involved: (i) an alerting network, involved in producing and maintaining adequate levels of activation in the cognitive systems required for efficient task performance; (ii) an orienting network for prioritising sensory input and selecting signals for focal processing; and (iii) an executive network involved in target detection (i.e. focal attention) and task maintenance (Petersen and Posner, 2012).

The alerting network is typically studied with paradigms involving a warning signal indicating that a target stimulus will appear (phasic alertness) or by using a long and monotonous task to measure sustained attention, or vigilance (tonic alertness). The neural correlates of the alerting network include brain stem arousal systems, particularly the noradrenergic system arising from the locus coeruleus, and right hemisphere systems relating to vigilance. The orienting function appears to rely on a network of frontal, parietal and subcortical regions. Specifically, cholinergic systems arising in the basal forebrain, which have been linked to delirium and acute cognitive dysfunction in animals (Field *et al.*, 2012), appear to play a critical role in orienting. Further, human and animal studies of orienting have suggested a role for the frontal eye fields, which are involved in eye movements and control of visual attention (Corbetta and Shulman, 2002). Interestingly, one study showed that eye movements, particularly blinks, were affected in delirium (van der Kooi *et al.*, 2014). The executive attention system involves a complex functional and anatomical network, specialising in target detection, focused attention and sustained activity related to maintaining task parameters and top‐down control (Petersen and Posner, 2012). There is much overlap among the constructs of executive attention, working memory and fluid intelligence (Tieges *et al.*, 2014).

Attentional deficits in delirium range from a state of lowered LoA, whereby patients may be unable to respond to simple commands, to deficits on tasks of orienting and selective attention and finally to more subtle impairments in complex cognitive tasks. These variations in inattention occur between patients but also often within the same patient at different time points. Therefore, it is implausible that a single neural mechanism can explain the diverse manifestations of inattention in delirium. More likely is that the various causes of delirium affect several neural systems underpinning attention.

Because the alerting and orienting networks form necessary components for higher‐order cognitive functions, disturbances in these networks result in widespread impairment, or at least our ability to measure these domains objectively (Leonard *et al.*, 2016). Thus, an apparent profile of broad deficits may actually result from impairments in more restricted core processes. In turn, higher‐order cognitive processes may be particularly susceptible to the effects of pathophysiologies other than delirium, and consequently, impairments in these more complex cognitive functions may become less specific for delirium.

### Studies of attentional deficits in delirium

Studies have used a range of tests for measuring attention in delirium including vigilance tests, the months of the year backward (MOTYB) test and counting up tasks (e.g. Adamis *et al.*, 2016; Brown *et al.*, 2011; Meagher *et al.*, 2010; O'Keeffe and Gosney, 1997; O'Regan *et al.*, 2014). Most of these tests involve different types of attention and differ in terms of the demands placed on other cognitive, overlapping processes including (working) memory and executive function (Corbetta and Shulman, 2002). Indeed, multiple cognitive systems including language, memory, perceptual, motor and executive functions may contribute to neuropsychological test performance.

For instance, vigilance tests provide a relatively ‘pure’ measure of attention, whereas backward span tests and MOTYB measure multiple cognitive domains (Meagher *et al.*, 2015). The majority of studies have reported attentional deficits in delirium across different populations (Brown *et al.*, 2011; Meagher *et al.*, 2010; Rajlakshmi *et al.*, 2013).

Most attention tests used in the assessment of delirium are sensitive to delirium. As stated previously, this may be because lower levels of attention, notably sustained attention and orienting, are a prerequisite for higher levels of attentional and cognitive functioning (Petersen and Posner, 2012). Nevertheless, there is some evidence supporting the view that sustained attention is disproportionately affected in delirium (Brown *et al.*, 2011; Tieges *et al.*, 2014, 2015). Studies that have compared patients with delirium and dementia suggest that attentional deficits are often greater in delirium but with varying degrees of overlap in scores (Brown *et al.*, 2011; Leonard *et al.*, 2016; Lowery *et al.*, 2008; Tieges *et al.*, 2015). Indeed, some studies have shown impairments on bedside tests of attention in dementia, including spatial span (Meagher *et al.*, 2010), serial 7s (Bronnick *et al.*, 2007) and MOTYB (Meagher *et al.*, 2015). Voyer *et al.* (2016) administered the MOTYB to older hospitalised patients with and without cognitive impairment, some of whom had delirium. MOTYB showed high sensitivity to delirium but poor specificity, because of impaired performance by patients with cognitive impairment but without delirium. In contrast, a purer measure of sustained visual attention assessed with a computerised instrument (Edinburgh Delirium Test Box) or a smartphone version of the same test (DelApp) was impaired in delirium but mostly intact in dementia (Brown *et al.*, 2011; Tieges *et al.*, 2015).

In addition to dementia, mood and psychotic disorders also share common features with delirium including inattention (Godard *et al.*, 2011; Gonzalez‐Blanch *et al.*, 2012; Lee *et al.*, 2012). Two studies compared performance on attention tests between patients with delirium and those with mood or psychotic disorder (Hart *et al.*, 1996; Trzepacz *et al.*, 1988). Hart *et al.* (1996) found that ICU patients with delirium performed worse on tests of attention compared with psychiatric inpatients with depression or schizophrenia (although differences in disease severity may have played a role). Another study (Trzepacz *et al.*, 1988) reported impaired Trail Making Test performance in patients with delirium and schizophrenia, but importantly less than half of delirious patients could attempt the test.

To conclude, delirium, depression and psychotic disorders have overlapping features, but delirium disturbances of attention appear to be more severe.

In summary, the neuropsychological literature shows that attention is impaired in delirium. Comparison of study findings is hampered by variations in the type of attention and other cognitive domains required for task performance, delirium severity, the type and severity of the mental disorder in the comparison groups and the patient population and setting. Caution is warranted when interpreting findings from attention tests, because these usually give participants a specific task (e.g. memorising digit sequences) and therefore also require other cognitive processes. Further, whether a test is labelled as an ‘attention test’ often depends on the theoretical context in which it is used (Meagher *et al.*, 2015). Another issue is that many of the instruments for assessing attention in delirium have not been rigorously validated in older patient populations. Notably, floor effects and failures to attempt tests have been reported (e.g. Christensen *et al.*, 1996; Trzepacz *et al.*, 1988). These may be partly due to problems with task understanding or initiating tasks, rather than inattention per se.

The evidence suggests that cognitive tests that rely on focusing and sustaining attention rather than requiring manipulation of information or testing memory may be especially useful in delirium research and clinical practice, particularly for discriminating delirium from dementia. Indeed, the ability to focus and sustain attention appears to be relatively preserved in the earlier stages of Alzheimer's dementia (Perry and Hodges, 1999).

## Level of arousal

Level of arousal, also termed ‘level of consciousness’, ‘wakefulness’, ‘somnolence’ or ‘sedation’, is even less well studied than attention in the context of delirium (Chester *et al.*, 2012; Meagher *et al.*, 2008; Ross *et al.*, 1991). LoA refers to the global level of behavioural responsiveness and relates to the degree of sensory stimulation required to keep a person awake and attentive (Posner *et al.*, 2007).

Level of arousal and attention are generally regarded as hierarchically related, whereby LoA must be sufficient before attention can be formally tested and impaired arousal impacts all other cognitive domains (European Delirium Association and American Delirium Society, 2014). In support of this view, a strong association between abnormal LoA and objectively measured deficits in sustained attention has been reported (Tieges *et al.*, 2013).

Level of arousal is assessed distinct from attentional or other cognitive deficits in several delirium scales, such as the 4 A's Test (Bellelli *et al.*, 2014), the Confusion Assessment Method (CAM; Inouye *et al.*, 1990), the Delirium Index (McCusker *et al.*, 1998) and the Memorial Delirium Assessment Scale (MDAS; Breitbart *et al.*, 1997). LoA is also measured using standalone observational scales including the Richmond Agitation Sedation Scale (Sessler *et al.*, 2002), the modified Richmond Agitation Sedation Scale (Chester *et al.*, 2012) and the Observational Scale for Level of Arousal (Tieges *et al.*, 2013).

Recent studies using these scales have suggested that abnormal LoA has high specificity for delirium in older patients (Chester *et al.*, 2012; Tieges *et al.*, 2013; Han *et al.*, 2015; Morandi *et al.*, 2016). Thus, acute alterations in LoA strongly indicate delirium, although because this feature is not always present, its sensitivity is lower.

Altered LoA could be particularly useful for detecting delirium in patients with chronic cognitive disorders. This is because arousal is generally preserved in these disorders until the later stages, possibly because the brainstem structures involved in regulating LoA are generally not affected in mild‐to‐moderate disease stages (Leonard *et al.*, 2016; Morandi *et al.*, 2017). In contrast, cognition is often impaired in more advanced dementia to the point where patients cannot engage with detailed formal testing (Kolanowski *et al.*, 2012). Of note, altered LoA or sundowning is commonly reported in severe dementia (Bachman and Rabins, 2006). Therefore, assessment of abnormal LoA may be less useful for detecting delirium in advanced dementia.

To conclude, the available evidence suggests that acute‐onset altered LoA is a specific marker for delirium; in clinical practice, abnormal LoA should be used as a trigger for delirium assessment.

## Disorganised thinking

Disorganised thinking indicates a disturbance of the organisation and expression of thought. It is common in psychotic disorders and is sometimes present in delirium (Young *et al.*, 2011). It was listed in the DSM‐III‐R criteria for delirium (American Psychiatric Association, 1987) but was no longer included from 1994 onwards (DSM‐IV), possibly because disorganised thinking is difficult to define and operationalise. Manifestations are diverse and include slowing down or speeding up of speech, impaired capacity to make judgments or grasp abstract concepts or loose associations (Burns *et al.*, 2004). Disorganised thinking is variably assessed in delirium tools, including subjective judgement following interview (CAM, Inouye *et al.*, 1990), Delirium Rating Scale‐Revised 98 (DRS‐R98; Trzepacz *et al.*, 2001), MDAS (Breitbart *et al.*, 1997), logical questions (CAM for the ICU, Ely *et al.*, 2001) and orientation (3D‐CAM; Marcantonio *et al.*, 2014).

Few studies have examined the different elements of disorganised thinking in delirium using neuropsychological tests, and data on psychometric parameters are mostly lacking. Its construct validity has been questioned with some authors arguing that its multiple characteristics can better be explained as the product of other impairments in delirium, including arousal, attention and memory deficits (Bhat and Rockwood, 2007). In contrast, the core features of altered arousal and inattention cannot be similarly reduced. Of interest, it has been proposed that a breakdown in selective attention could explain most of, if not all, the symptoms of disturbed thought in schizophrenia (Lake, 2008).

To conclude, disorganised thinking requires further research on its characteristics and the extent to which its variable features can be explained by deficits in underlying cognitive processes such as inattention.

## Language

Language dysfunction comprises impairments in communicating through speech or writing (expressive aphasia) and difficulty understanding spoken or written language (receptive aphasia). The DSM‐5 criteria for delirium include language disturbance, but methods of assessment are not specified. There are very few studies on language in delirium. Wallesch and Hundsalz (1994) reported group differences in a word naming and comprehension task. Another study found that writing disturbance (dysgraphia) was present in almost all patients with delirium (Chedru and Geschwind, 1972). Adamis *et al.* (2006) showed that an abnormal signature was specific (88%), but not sensitive, to delirium. Handwriting abnormalities were also more common in delirium, mainly because many delirious patients were unable to provide any handwritten response. Using the DRS‐R98, Meagher *et al.* (2007) identified language abnormalities in over half of delirious patients.

Language skills depend on a range of motor and cognitive functions including attention, working memory and visuospatial processing, and each of these may contribute to language impairments in delirium. This is analogous to the notion that disorganised thinking may in part be secondary to more fundamental cognitive dysfunctions. Better understanding of speech and language disturbances in delirium could aid diagnosis and increase awareness of speech production and comprehension difficulties in delirium, which may require adaptation of communication strategies to the respective needs of patients with delirium.

## Visual perception

Perceptual disturbances, such as hallucinations, misperceptions and illusions (particularly visual) are frequently present in delirium, suggesting that patients with delirium may have deficits in the cognitive systems underlying visual perception. This idea is in line with models of visual hallucinations, which postulate that a combination of perceptual and attention deficits can cause incoming sensory information to activate incorrect or irrelevant neural representations stored in memory and subsequently cause hallucinations or illusory misperceptions (Collerton *et al.*, 2005). Yet these potential underlying perceptual deficits have hardly been studied in delirium. Most knowledge about perceptual disturbances in delirium has come from subjective patient reports and clinical observation.

One study found that perceptual disturbances and impairments in visuospatial ability were present in, respectively, 50% and 87% of patients with delirium (Meagher *et al.*, 2007). Thus, visuospatial deficits, referring to the ability to perceive shapes, spatial relationships and details of figures or objects, may be particularly common in delirium (Trzepacz *et al.*, 2001). Indeed, there are many clinical accounts of fleeting visuospatial disturbances in delirium, which include alteration of position in space and gaps in the contours of objects (Lipowski, 1990). However, only two studies have quantified perceptual deficits in delirium using neuropsychological tests, finding specific impairments in visual perception worse than in controls or patients with dementia (Brown *et al.*, 2009; Leonard *et al.*, 2016).

Examination of perceptual deficits is a component of several delirium assessment tools. For instance, the DRS‐R98 includes items on perceptual disturbances and visuospatial ability assessed through interview, observation and cognitive testing (Trzepacz *et al.*, 2001). The Delirium Index assesses perceptual disturbances through patient interview questions (McCusker *et al.*, 2004).

In summary, perceptual deficits occur commonly in delirium but are understudied. The presence of perceptual disturbances in delirium contributes significantly to patient and caregiver distress (Breitbart *et al.*, 2002) and may underlie common problems of poor environmental interactions, wandering and falls in delirium (Meagher *et al.*, 2007). Better understanding of these deficits may help improve clinical care in delirium. The few cognitive studies currently available suggest that objective neuropsychological testing may be valuable in detecting and characterising visual perceptual deficits in delirium.

## Memory

Memory impairment is part of the current diagnostic criteria for delirium (Table 1) and is evaluated in several tools. Short‐term and long‐term memory deficits were common in palliative care patients with delirium (88% and 89%, respectively), although these deficits did not differ in presence or severity between groups with delirium and dementia (Meagher *et al.*, 2007, 2010). In contrast, Brown *et al.* (2009) found that older acute care hospital patients with delirium performed better on tests of verbal memory function compared with outpatients with dementia, although with worse performance than cognitively intact inpatients. It is unclear whether these memory deficits in delirium reflect primary memory dysfunctions or if they are secondary to attentional deficits, because intact attention is required to encode and recall new information.

In summary, memory impairment is common in delirium but appears to be non‐specific, and the extent to which memory problems reflect inattention in delirium is presently unclear.

## Other features of delirium

Features of delirium not covered in detail in this review include the acute onset and fluctuating course of symptoms (both core features of delirium), sleep–wake cycle disturbance, altered affect, disorientation and motor incoordination (Bellelli *et al.*, 2011). Establishing acute onset and fluctuation of features requires information beyond what can be elicited on a single bedside assessment, including information obtained from informants, records on pre‐admission functional status and case notes. Acute onset and fluctuations may also be revealed through repeated clinical observation and cognitive testing.

Motor disturbance is common in delirium, and clinical subtyping according to motor‐activity profile has received considerable interest in the literature (Grover *et al.*, 2014). Motor alterations of delirium are linked to changes in arousal and are included in several arousal scales (Sessler *et al.*, 2002; Tieges *et al.*, 2013). Although the associations among all these features are poorly understood, motor activity levels may be a useful proxy for altered arousal (Meagher, 2009).

## Discussion

The neuropsychological approach, involving explicit descriptions of psychological constructs, along with use of quantitative, objective instruments supported by formal psychometric analysis, has much to contribute in developing more reliable, robust and standardised assessments of the features of delirium. Yet relatively few studies have aimed to characterise and quantify the cognitive and behavioural features of delirium using objective methods (Hart *et al.*, 1996; Meagher *et al.*, 2010; Tieges *et al.*, 2014, 2015). Additionally, the psychometric performance of existing methods of determining the presence or absence of the features listed in these tools or criteria (such as inattention) has mostly not been examined in detail. Many methods rely on binary subjective judgements, which often show unsatisfactory inter‐rater reliability.

Most neuropsychological studies in delirium have focused on attentional function. These studies confirm that patients with delirium nearly always show impairments on objective tests of attention compared with cognitively intact controls and, in most cases, patients with dementia. Tests of focused and sustained attention appear to be particularly useful for differential diagnosis (Brown *et al.*, 2011). However, findings from dementia groups should be interpreted with caution, because dementia has several subtypes and levels of severity (Morandi *et al.*, 2012). The role of LoA in assessing delirium requires further study, but the available empirical evidence indicates that abnormal LoA is a strong and specific indicator of delirium (Han *et al.*, 2015).

The neuropsychological evidence base for other cognitive impairments in delirium is also limited. Some papers have reported deficits in visual perception, language and thought processes in delirium (Adamis *et al.*, 2006; Brown *et al.*, 2009). However, there is uncertainty regarding the nature of these impairments, the extent to which they are present in delirium and if they might contribute reliably to the diagnostic process.

An important conclusion arising from this review is that the component features of delirium lack explicit and agreed definitions, and terms describing these features are used inconsistently and loosely. Consensus on operationalisation and assessment methods of these symptom domains in delirium is also lacking. For example, questions such as ‘Does a stone float in water?’ have been used in delirium assessment instruments to measure ‘disorganised thinking’ (Ely *et al.*, 2001) but also ‘auditory comprehension’ (Hart *et al.*, 1996). There is also overlap in the definitions and operationalisations of other constructs including language and disorganised thinking; for example, poverty of speech can indicate the presence of both features. Such inconsistencies hamper comparisons among research studies and their eventual translation into clinical practice.

Several recommendations arise from this review. Firstly, the uncertainty with respect to the conceptualisation and measurement of the component features of delirium has critical implications for the delirium reference standard used in research. There is significant variability in reference standard methods for delirium diagnosis (Neufeld *et al.*, 2014), and it is mostly not specified how each delirium feature was assessed. We recommend more detailed and explicit documentation regarding the reference assessment process used, including specification of the methods employed to assess the individual features of delirium (for example Neerland *et al.*, 2015). Such an approach will facilitate comparison of research findings and help evaluate study quality. A common reference standard incorporating agreed assessment methods of the individual features of delirium and a standardised diagnostic algorithm would be of great value.

Secondly, the validity of neuropsychological testing of individual delirium features has limits, because these features vary widely between and within patients, and delirium shares neuropsychological deficits with other conditions such as dementia, depression and psychosis. Thus, eliciting the time course and pattern of deficits in relation to a person's baseline mental status is necessary for optimal diagnostic accuracy.

There are numerous gaps in our knowledge regarding the neuropsychology of delirium, including studies reporting on longitudinal cognitive data, usefulness of tests for discriminating delirium from other neurocognitive disorders including dementia, mood and psychotic disorders, test reliability and validity and the relationship between neuropsychological test scores with subjective measures or clinical outcomes, which will help clarify the prognostic value of tests. Additional research could lead to the provision of better tools for diagnosis, monitoring and severity grading in clinical practice and in research, for example in treatment trials. Delirium assessment tools should draw from existing best practice in neuropsychology but must also involve new methods and approaches that take account of the challenges of delirium assessment in real‐world practice. Many existing neuropsychological tests are time consuming and effortful and might not be acceptable to most patients with delirium. Moreover, fine‐grained measures of delirium for severity grading may be more suitable in clinical trials than dichotomous scoring methods for measuring and monitoring this fluctuating syndrome. However, severity in delirium is as yet not clearly defined.

### Conclusion

The neuropsychological evidence base in delirium remains limited, and studies are only beginning to unveil the complex neuropsychological profile of delirium. More research will lead to greater understanding of how constructs such as inattention, language impairment and disorganised thinking are inter‐related and defined. Ultimately, greater adoption of the neuropsychological approaches will aid the development of objective methods of assessment that are more reliable, valid and reproducible, allowing for greater comparability among studies.

## Conflict of interest

A. M. J. M. has patents on objective computerised assessments of attention.

**Key points**

- The component features of delirium lack explicit and agreed definitions, and there is no consensus on assessment methods of symptom domains.
- Most neuropsychological studies in delirium have focused on attention. The evidence base for impairments in other cognitive domains is limited.
- We recommend detailed, explicit documentation of the reference standard process used in future papers, allowing for greater study comparability.
- Greater adoption of the neuropsychological approach in delirium research will inform the development of more valid and reliable assessment methods.
